# Real-Time AI-Assisted Insulin Titration System for Glucose Control in Patients With Type 2 Diabetes: A Randomized Clinical Trial

**DOI:** 10.1001/jamanetworkopen.2025.8910

**Published:** 2025-05-07

**Authors:** Zhen Ying, Yujuan Fan, Congling Chen, Yuchen Liu, Qi Tang, Zhiwei Chen, Qian Yang, Hongmei Yan, Liming Wu, Jiaping Lu, Zhiwen Liu, Jun Liu, Xiaoying Li, Ying Chen

**Affiliations:** 1 Ministry of Education Key Laboratory of Metabolism and Molecular Medicine, Department of Endocrinology and Metabolism, Zhongshan Hospital, Fudan University, Shanghai, China; 2 Department of Endocrinology and Metabolism, Minhang Hospital, Fudan University, Shanghai, China; 3 Department of Geriatrics, Zhongshan Hospital, Fudan University, Shanghai, China; 4 Department of Endocrinology and Metabolism, Guangdong Provincial Key Laboratory of Diabetology, The Third Affiliated Hospital of Sun Yat-sen University, Guangzhou, Guangdong, China; 5 Big Data and Artificial Intelligence Center, Zhongshan Hospital, Fudan University, Shanghai, China; 6 Department of Endocrinology, Fifth People’s Hospital of Shanghai, Fudan University, Shanghai, China; 7 Department of Endocrinology, XuHui Central Hospital of Shanghai, Shanghai, China; 8 Department of Endocrinology and Metabolism, Qingpu Branch of Zhongshan Hospital Affiliated to Fudan University, Shanghai, China

## Abstract

**Importance:**

Type 2 diabetes (T2D) is one of the most prevalent chronic diseases in the world. Insulin titration for glycemic control in T2D is crucial but limited by the lack of personalized and real-time tools.

**Objective:**

To examine whether an artificial intelligence–based insulin clinical decision support system (iNCDSS) for glycemic control in hospitalized patients with T2D is noninferior to standard insulin therapy administered by senior physicians.

**Design, Setting, and Participants:**

This multicenter, single-blind, parallel randomized clinical trial (RCT) was conducted between October 1, 2021, and September 8, 2022, in endocrinology wards of 3 medical centers. Eligible participants were adults (aged ≥18 years) with glycated hemoglobin levels between 7.0% and 11.0% who had received antidiabetic treatments in the previous 3 months.

**Interventions:**

Participants were randomized in a 1:1 ratio to receive insulin dosage titration by iNCDSS or senior endocrinology physicians for 5 consecutive days.

**Main Outcomes and Measures:**

The primary outcome was the proportion of time in the target glucose range (70-180 mg/dL) during the 5-day study period; the noninferiority margin was 6 percentage points. Secondary outcomes included other glycemic control measurements and adverse events.

**Results:**

A total of 149 participants (mean [SD] age, 64.2 [12.0] years; 84 male [56.4%]) were enrolled and randomized to the iNCDSS group (n = 75) or physician group (n = 74). The mean (SD) target glucose range (primary outcome) was 76.4% (16.4%) in the iNCDSS group and 73.6% (16.8%) in the physician group, which achieved the prespecified noninferiority criterion (estimated treatment difference, 2.7%; 95% CI, −2.7% to 8.0%). There were no significant differences in adverse events between the 2 groups. Most physicians were satisfied with the iNCDSS for its clear, time-saving, effective, and safe clinical support.

**Conclusions and Relevance:**

In this RCT of an iNCDSS, the system demonstrated noninferiority to senior endocrinology physicians in insulin titration in an inpatient setting, indicating its potential as a favorable tool for insulin titration in patients with T2D.

**Trial Registration:**

ClinicalTrials.gov Identifier: NCT04642378

## Key Points

**Question:**

Can an artificial intelligence–based insulin clinical decision support system for type 2 diabetes achieve glycemic outcomes that are not inferior to standard therapy administered by senior endocrinology physicians?

**Findings:**

In this randomized clinical trial of 149 adults with type 2 diabetes, the glycemic control of the artificial intelligence–based insulin clinical decision support system intervention was not inferior to that of standard therapy by senior physicians, without increasing risk of adverse events.

**Meaning:**

The findings of this trial suggest that an artificial intelligence–based digital tool can achieve noninferior or possibly better outcomes compared with standard therapy by providing real-time and personalized clinical support.

## Teaser

This randomized clinical trial of adults with type 2 diabetes assesses the efficacy and safety of an artificial intelligence–based insulin clinical decision support system in achieving glucose control.

## Introduction

Type 2 diabetes (T2D) is one of the most prevalent chronic diseases and leads to a considerable rate of death and social burden worldwide.^1^ Many adults with T2D eventually require insulin therapy,^2^ and the total volume of insulin required to treat T2D is expected to increase by more than 20% from 2018 to 2030.^3^ However, less than one-third of patients with T2D treated with insulin achieve their diabetes management goals.^4,5^ This suboptimal glycemic control is attributed partly to the prescription complexity, clinical inertia, and fear of hypoglycemia in insulin titration.^6,7,8^ Although a series of clinical guidelines on rational insulin use for patients with T2D has been proposed by experts,^2,9,10,11^ the guidelines cannot fully take into consideration the heterogeneity of each patient.^12,13^ Moreover, traditional insulin adjustment faces constraints as diabetes prevalence increases and specialized endocrinologists remain scarce.

Several digital technologies have emerged to support the management of glucose control, including dose calculations, insulin titration algorithms based on artificial intelligence (AI), and closed-loop insulin delivery systems.^14^ However, only a limited number of approaches consider the diverse and dynamic characteristics of T2D, and most of them are still in the preclinical simulation phase.^15,16^ In addition, current solutions were mainly designed for single insulin regimen,^17,18^ which limits their applications in patients with different treatments. Therefore, there is a critical need for a personalized insulin decision-making tool that is safe, effective, and broadly applicable for T2D.

Previous work^19^ developed an AI-based insulin clinical decision support system (iNCDSS) to optimize insulin treatment. Leveraging personalized algorithms, the iNCDSS outperformed other models and matched senior physicians’ performance in a retrospective dataset. After integrating this tool into clinical practice, a proof-of-concept trial demonstrated the feasibility of our tool in improving glucose control without increasing the risk of hypoglycemia.

This study assesses the efficacy and safety of iNCDSS in patients with T2D receiving insulin therapy in endocrinology wards. We hypothesized that iNCDSS would be noninferior to standard insulin therapy by senior endocrinology physicians in an inpatient setting.

## Methods

### Study Design

This trial was a multicenter, single-blind, parallel randomized clinical trial (RCT) that evaluated the efficacy and safety of iNCDSS in hospitalized patients with T2D. The trial was completed at 3 medical centers in China: Zhongshan Hospital, Xuhui Central Hospital, and Shanghai Fifth People’s Hospital. The study was approved by the ethical committees at each center. Each participant provided written informed consent. This study was reported in concordance with the Consolidated Standards of Reporting Trials (CONSORT) reporting guideline. The trial protocol can be found in Supplement 1.^20^

### Participants

Between October 1, 2021, and September 8, 2022, a total of 305 individuals were screened. Eligible participants were adults (aged ≥18 years) diagnosed with T2D with glycated hemoglobin (HbA_1c_) levels between 7.0% and 11.0% (to convert to proportion of total hemoglobin, multiply by 0.01) who had been treated with diet, any combination of oral antidiabetic agents, and/or insulin therapy in the previous 3 months. These patients with poorly controlled T2D were admitted to endocrinology wards for glycemic control.

Key exclusion criteria were acute complications of diabetes; body mass index of 45 or greater (calculated as weight in kilograms divided by height in meters squared); pregnancy or breastfeeding; severe cardiac, hepatic, or kidney diseases; psychiatric or psychological diseases; severe edema, infections, or peripheral circulation disorders; and surgery during hospitalization. A full list of exclusion criteria is included in the trial protocol (Supplement 1).

### Randomization and Masking

Eligible participants were randomly assigned (1:1) to the iNCDSS group or physician group using block randomization with varying block sizes of 4 to 6. Randomization was stratified by sites and baseline HbA_1c_ values (<8.0% or ≥8.0%). The randomization sequence was generated by an independent qualified statistician. Treatment allocation was masked from participants and was concealed with sealed opaque envelopes.

### Procedures

**Figure 1.**
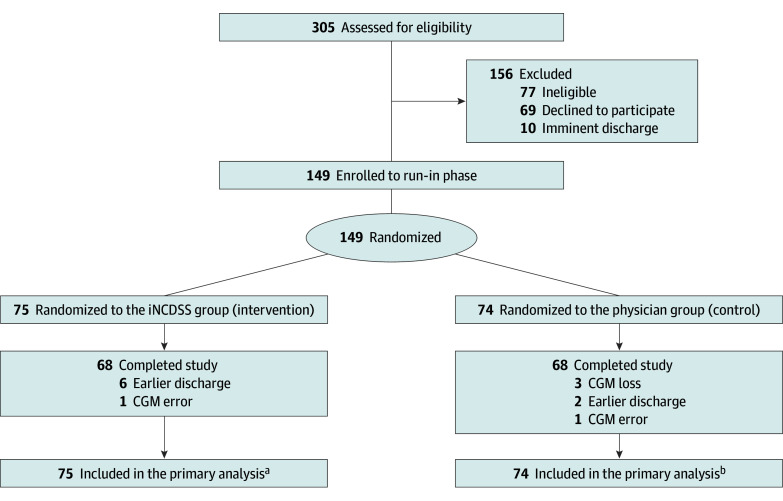
CONSORT Diagram CGM indicates continuous glucose monitoring; iNCDSS, insulin clinical decision support system. ^a^The iNCDSS group had 74 participants with the primary outcome. ^b^The physician group had 70 participants with the primary outcome.

The trial consisted of screening, run-in, and randomized treatment phases (Figure 1). For each recruited participant, demographic and clinical characteristics were assessed for eligibility. After the screening, eligible participants entered a run-in phase during which all participants received insulin therapy for a whole day. The initiation or switch of insulin regimen, if needed, was determined by endocrinologists based on patients’ glycemic control and guidelines. All other antidiabetes medications were continued except sulfonylurea (eMethods in Supplement 2). For each patient, capillary glucose concentrations were measured before meals and bedtime using a glucometer (Glupad [Sino Medical Sciences Technology Inc]), and the glucose values were automatedly uploaded into the hospital information system for guiding insulin dosage.

Eligible participants were randomly assigned to the intervention or control group. Participants in the intervention group received insulin dosage titration based on the recommendations of the iNCDSS tool, whereas those in the control group received insulin dosage titration determined by senior physicians (Q.Y., L.W., and H.Y.), all of whom were board-certified attending physicians with more than 10 years of experience in endocrinology. Both groups were studied for 5 consecutive days.

The process in the intervention group administered by the iNCDSS has been described previously.^19^ The model was implemented into the clinical settings to build a workflow for insulin titration support, which could read in real time the patient information and provide insulin recommendations in the interface (eMethods in Supplement 2). The insulin titration recommendation covered 3 common subcutaneous insulin regimens (basal insulin only, biphasic or premixed insulin regimen, and basal-bolus regimen). After the warm-up of the first run-in day, the algorithm automatically read the insulin regimen and calculated an updated insulin regimen, providing precise insulin dosage recommendations for the subsequent 24 hours. These recommendations were subsequently reviewed and confirmed by the physicians during their daily morning rounds. Physicians’ adherence to and deviations from recommended insulin doses were recorded. Physicians who used the iNCDSS tool completed a questionnaire to evaluate their satisfaction with the tool (eMethods in Supplement 2). In the control group, insulin dosage was prescribed and titrated by senior physicians (Q.Y., L.W., and H.Y.) based on capillary blood glucose measurements and clinical information in accordance with clinical practice.

Throughout the intervention period, continuous glucose monitoring (CGM) was performed using flash glucose monitoring (Freestyle Libre [Abbott]) in both groups. Participants did not have access to CGM data, and physicians did not use CGM data to make any treatment decisions. The antidiabetic medications were not changed during the intervention period. Participants in both groups were provided standard meals at the usual mealtime. No restrictions were placed on consuming other meals and snacks or on usual activities.

### Outcomes

The primary outcome was the proportion of time in the target glucose range (TIR; 70-180 mg/dL) measured by CGM during the 5-day intervention period. Secondary outcomes included the proportion of time in which the glucose level was above the target range (180-250 or >250 mg/dL) or below the target range (54-70 or <54 mg/dL), mean sensor glucose concentration, glucose management indicator, glucose variability (defined as the coefficient of variation of sensor glucose), mean premeal and prebed capillary glucose concentration, and the mean total daily insulin dose during the 5-day intervention period. Secondary outcomes were analyzed without predefined noninferiority margins and therefore were interpreted as exploratory. Daytime (8 am to midnight) and overnight (midnight to 8 am) results were calculated for a subset of outcomes (proportion of time in, below, or above the target glucose range, mean sensor glucose measurement, and the coefficient of variation in sensor glucose measurement, with the use of data from the respective periods) to limit multiple comparisons. Assessment of the satisfaction of physicians in the iNCDSS group was collected at the end of the study using a questionnaire with a 5-point Likert scale from 1 to 5, with 1 indicating very dissatisfied or disagree and 5 indicating very satisfied or agree (eMethods in Supplement 2). A full list of all secondary and other prespecified outcomes is in the protocol (Supplement 1). Safety outcomes include the incidence of clinically significant hypoglycemia (glucose <54 mg/dL), severe hypoglycemia (glucose <40 mg/dL or an episode that required the assistance of another person), and clinically significant hyperglycemia (glucose >360 mg/dL) or ketonemia, along with other adverse events and serious adverse events.

### Statistical Analysis

We hypothesized that the use of the iNCDSS would not be inferior to senior physician recommendations of the TIR (70-180 mg/dL). According to previous studies,^19,21,22^ the mean (SD) TIR (70-180 mg/dL) was 80% (12%), and a noninferiority margin of 6 percentage points was chosen to assess the difference between the groups. We calculated a sample size of 142 participants (considering a dropout rate of approximately 10%) to achieve a power of 80% with an α = .025 (1-sided 95% CI).

In the RCT, we analyzed efficacy and safety data by intention to treat. This primary analysis included all randomized study participants, and participants were analyzed according to the treatment they were assigned. We also conducted a per-protocol analysis. The primary and secondary outcomes were compared with a linear mixed-effect regression model with treatment (iNCDSS and physician group) as the fixed effect, site as a random effect, and baseline HbA_1c_ level as a covariate. The noninferiority hypothesis was tested for the primary outcome by estimating the difference in TIR of 70 to 180 mg/dL between the 2 groups, and the lower limit of the 95% CI of −6.0% was set as the noninferiority threshold. Additional analysis was performed with baseline insulin dosage as a covariate included in the linear mixed-effect regression models. Multiple imputation was also used to impute the missing data of the primary outcome to determine whether the result was consistent. We tabulated the numbers of events (safety outcome) that were related to a capillary glucose measurement of less than 54 mg/dL, less than 40 mg/dL, or more than 360 mg/dL and ketonemia in each group, and we compared the proportion of participants with events between the 2 groups with the Fisher exact test. We report values as means (SDs) or medians (IQRs) unless stated otherwise. All statistical analyses were performed using R version 4.3.2 (R Project for Statistical Computing) and SAS version 9.3 (SAS Institute). Data analysis was performed from November 2023 to March 2024.

## Results

### Study Participants

Of the 305 individuals screened, 149 were eligible and consented. The mean (SD) age of the study population was 64.2 (12.0) years, 65 (43.6%) were female, and 84 (56.4%) were male. Mean (SD) body mass index was 25.1 (3.9), and mean (SD) HbA_1c_ level at enrollment was 9.0% (1.1%) (eTable 1 in Supplement 2). Of the 149 hospitalized participants, 75 were randomly assigned to the iNCDSS group and 74 to the physician group. Thirteen participants discontinued the study before completion: 7 from the iNCDSS group (6 due to earlier discharge and 1 due to CGM error) and 6 from the physician group (2 due to earlier discharge, 1 due to CGM error, and 3 due to CGM loss). Of these participants, 8 provided available CGM data for calculating the primary outcome (Figure 1). The primary analysis based on the intention-to-treat population included 75 participants in the iNCDSS group and 74 in the physician group. The demographics, baseline laboratory tests, disease conditions, and medication use of the 2 groups were similar (Table 1; eTable 2 in Supplement 2). The baseline characteristics by site are presented in eTable 3 in Supplement 2.

**Table 1.** Demographics and Baseline Characteristics of the Study Sample

Characteristic	No. (%) of participants	
	iNCDSS group (n = 75)	Physician group (n = 74)
Sex		
Male	45 (60.0)	39 (52.7)
Female	30 (40.0)	35 (47.3)
Age, mean (SD), y	63.4 (12.3)	65.0 (11.7)
BMI, mean (SD)	25.2 (4.1)	25.0 (3.7)
HbA_1c_, mean (SD), %	9.0 (1.1)	9.0 (1.0)
Duration of diabetes, mean (SD), y	13.1 (9.0)	15.9 (8.9)
Previous diabetes treatments		
Diet alone	0	1 (1.4)
Oral agents	30 (40.0)	20 (27.0)
Insulin alone	5 (6.7)	7 (9.5)
Insulin plus oral agents	40 (53.3)	46 (62.2)

Abbreviations: BMI, body mass index (calculated as weight in kilograms divided by height in meters squared); HbA_1c,_ glycated hemoglobin; iNCDSS, insulin clinical decision support system.

SI conversion factor: To convert HbA_1c_ to proportion of total hemoglobin, multiply by 0.01.

### Outcomes

The mean (SD) TIR (70-180 mg/dL [to convert to millimoles per liter, multiply by 0.0555]) was 76.4% (16.4%) in the iNCDSS group and 73.6% (16.8%) in the physician group. The lower bound of the 2-sided 95% CI for the difference in proportions between the 2 groups achieved the prespecified noninferiority criterion of −6% (difference, 2.7%; 95% CI, −2.7% to 8.0%; *P* = .33) (Table 2). Consistent results were observed in the per-protocol analysis, which included 68 participants in each group (eTables 4 and 5 in Supplement 2). Results of the linear mixed-effect regression model with baseline insulin dosage as a covariate and multiple imputation for handling missing data are provided in eTable 6 in Supplement 2.

**Table 2.** Glucose Control and Mean Daily Insulin Dose in the Randomized Clinical Trial

	iNCDSS group (n = 75)	Physician group (n = 74)	Estimated treatment difference (95% CI)	*P* value
Time in which the glucose level is within range, mean (SD), %^a^				
70-180 mg/dL	76.4 (16.4)	73.6 (16.8)	2.7 (−2.7 to 8.0)	.33
181-250 mg/dL	15.7 (11.3)	18.2 (12.0)	−2.5 (−6.3 to 1.3)	.20
>250 mg/dL	4.0 (7.7)	4.2 (8.2)	−0.2 (−2.8 to 2.4)	.87
54-70 mg/dL	3.3 (4.4)	3.1 (5.0)	0.2 (−1.3 to 1.8)	.77
<54 mg/dL	0.0 (0.0-0.4)	0.0 (0.0-0.6)	−0.2 (−0.9 to 0.4)	.52
Glucose, mean (SD), mg/dL^a^	139.9 (27.6)	144.4 (27.4)	−4.5 (−13.5 to 4.4)	.33
CV of glucose, mean (SD), %^a^	31.2 (7.0)	30.4 (6.8)	0.8 (−1.4 to 3.0)	.49
GMI, mean (SD), %^a^	6.7 (0.7)	6.8 (0.7)	−0.1 (−0.3 to 0.1)	.33
Capillary glucose concentrations, mean (SD), mg/dL				
Prebreakfast (5-8 am)	131.1 (28.0)	142.1(35.8)	−10.9 (−21.0 to −0.8)	.04
Prelunch (11 am-1 pm)	151.0 (47.8)	158.2 (46.9)	−6.2 (−19.6 to 7.2)	.37
Predinner (4-6 pm)	167.7 (45.3)	181.9 (47.3)	−13.8 (−28.3 to 0.8)	.07
Prebed (8-10 pm)	168.6 (49.1)	176.9 (48.3)	−7.3 (−22.4 to 7.9)	.35
Daily insulin dose, median (IQR), units	27.2 (20.0-34.4)	30.2 (22.8 to 42.6)	−5.4 (−9.3 to −1.6)	.01

Abbreviations: CV, coefficient of variation; GMI, glucose management indicator; iNCDSS, insulin clinical decision support system.

SI conversion factor: To convert glucose to millimoles per liter, multiply by 0.0555.

^a^ Participants who had available continuous glucose monitoring data were included in the analysis (74 participants in the iNCDSS group and 70 participants in the physician group).

**Figure 2.**
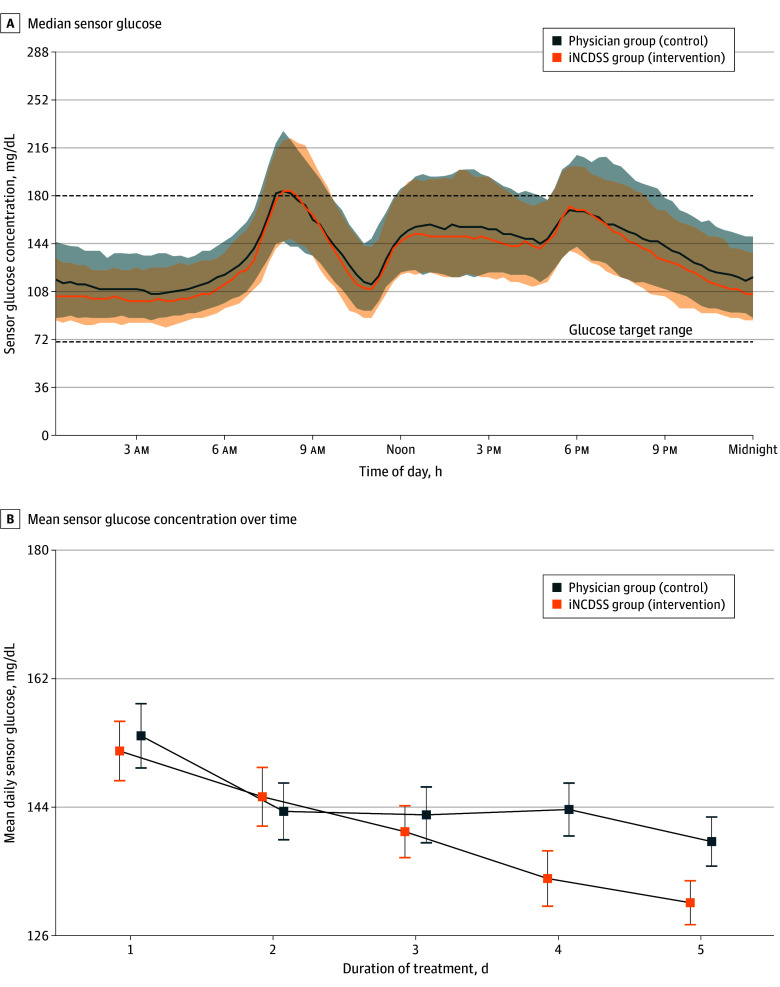
Sensor Glucose Concentration During Intervention A, Median sensor glucose concentrations. Solid orange line (orange shaded area) indicate median (IQR) sensor glucose concentration during use of the insulin clinical decision support system (iNCDSS); solid blue line (blue shaded area) indicate median (IQR) number of physician interventions. The brown shaded area indicates overlapping IQRs. The lower and upper limits of the target glucose concentration range of 70 to 180 mg/dL are denoted by the horizontal dashed lines. B, Mean sensor glucose concentration over time among participants who received iNCDSS (orange line) and senior physician (blue line) insulin recommendations. Error bars indicate standard error of the mean. To convert glucose to millimoles per liter, multiply by 0.0555.

The proportions of time in which the glucose level was above 250 mg/dL, within 180 to 250 mg/dL, within 54 to 70 mg/dL, or below 54 mg/dL as well as the coefficient of variation of glucose, glucose management indicator, and mean sensor glucose concentration, are given in Table 2. There were no statistically significant differences between the groups in these prespecified continuous glucose monitoring measurements. The 24-hour sensor glucose concentrations of the iNCDSS group and the physician group shown in Figure 2A indicate similar glucose fluctuations in both groups. In the iNCDSS group, the mean daily sensor glucose gradually decreased during the 5-day intervention period (Figure 2B). The mean (SD) prebreakfast capillary glucose in the iNCDSS group (131.1 [28.0] mg/dL) was significantly lower than that in the physician group (142.1 [35.8] mg/dL; *P* = .04), and the capillary glucose levels of prelunch (151.0 [47.8] in the iNCDSS group and 142.1 [35.8] mg/dL in the physician group; *P* = .04), predinner (167.7 [45.3] mg/dL in the iNCDSS group and 181.9 [47.3] mg/dL in the physician group; *P* = .07), and prebed (168.6 [49.1] mg/dL and 176.9 [48.3] mg/dL in the physician group; *P* = .35) were similar between the 2 groups (Table 2). The median (IQR) daily insulin dosage in the iNCDSS group was significantly lower (27.2 [20.0-35.5] units) compared with the physician group (30.2 [22.8-42.6] units; *P* = .01) (Table 2). However, when baseline insulin dosage was included in the model as a covariate, no significant difference of median daily insulin dosage was observed, suggesting that the observed between-group difference was primarily due to different baseline dosages rather than a reduction in adjustments by the iNCDSS (eTable 6 in Supplement 2).

Additionally, the TIR (70-180 mg/dL) was numerically higher in the iNCDSS intervention group compared with the physician group across different insulin regimens with no significant differences (basal insulin regimen: 78.6% [17.8%] vs 65.0% [40.2%]; premixed or biphasic insulin regimen: 74.7% [17.0%] vs 74.5% [14.9%]; basal bolus insulin regimen: 81.1% [9.7%] vs 73.3% [15.0%]) (eTable 7 in Supplement 2). Subgroup analyses were conducted in patients with or without prior insulin treatment as well as in patients with different levels of HbA_1c_. These findings demonstrated no statistically significant differences in glycemic control between the iNCDSS and physician groups across all these subgroups (eTables 8 and 9 in Supplement 2).

The proportion of time that overnight and daytime sensor glucose concentration was in the target range (70-180 mg/dL) was not significantly higher in the iNCDSS group vs the physician group (overnight: 84.9% [15.1%] vs 81.6% [17.9%]; difference, 3.3%; 95% CI, −2.0% to 8.6%; *P* = .23; daytime: 72.0% [20.6%] vs 69.6% [20.1%]; difference, 2.4%; 95% CI, −4.2% to 8.9%; *P* = .48). In addition, no significant differences were noted in mean sensor glucose, glucose management indicator, and glucose variability during daytime and overnight (eTable 10 in Supplement 2).

**Figure 3.**
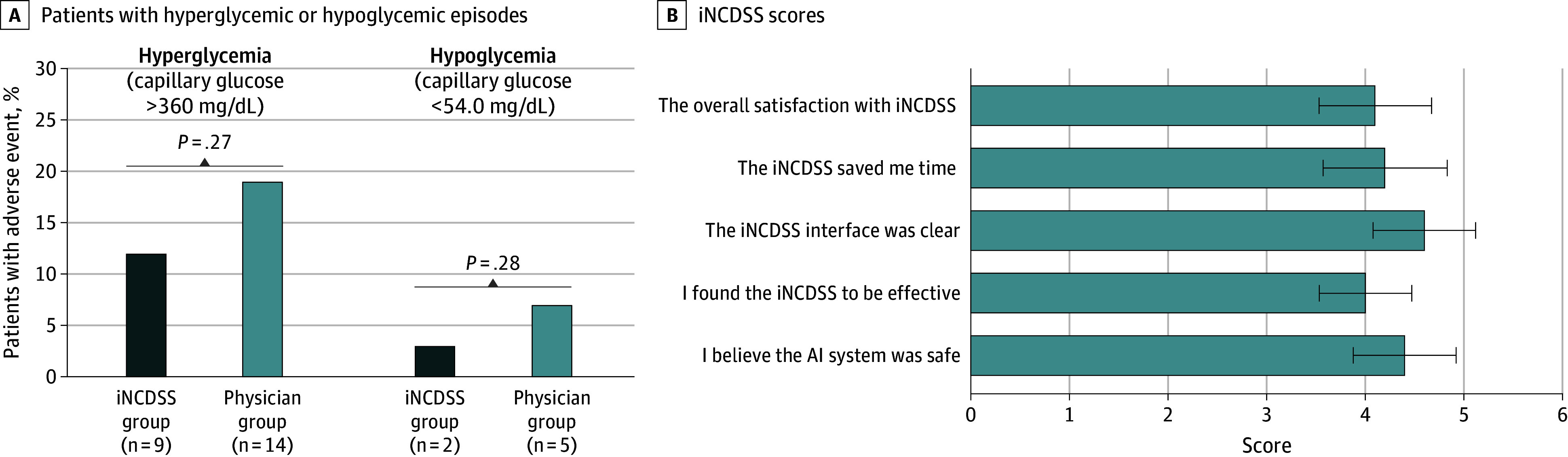
Adverse Events and Physicians’ Satisfaction A, Proportion of patients with hyperglycemic (>360 mg/dL) or hypoglycemic (<54 mg/dL) episodes. The number of patients with adverse events are shown below the x-axis. There were no statistically significant differences between the insulin clinical decision support system (iNCDSS) group and the physician group. B, Mean scores on the postintervention evaluation of the iNCDSS during the treatment trial assessed by physicians (n = 10) using questionnaires (eMethods in Supplement 2). The satisfaction agreement was scored on a scale of 1 to 5, with 1 indicating very dissatisfied or disagree and 5 indicating very satisfied or agree. Error bars indicate SDs. AI indicates artificial intelligence. To convert glucose to millimoles per liter, multiply by 0.0555.

The numbers of adverse events are shown in Figure 3A and eTable 11 in Supplement 2. There were no differences in the number of patients with capillary glucose levels greater than 360 mg/dL or capillary glucose levels greater than 54 mg/dL between groups by capillary glucose measurements (Figure 3A). No episodes of severe hypoglycemia with glucose levels less than 40 mg/dL or ketoacidosis occurred in either group. One adverse event of subcutaneous bruise related to CGM wearing was observed in the iNCDSS group (eTable 11 in Supplement 2).

During the intervention, 752 of 760 iNCDSS recommendations (98.9%) were adopted by physicians. Satisfaction questionnaires were completed by 10 physicians at the bedside who used our system in the trial. Most physicians stated that the iNCDSS interface was clear (4.6 of 5.0), time-saving (4.2 of 5.0), effective (4.0 of 5.0), and safe (4.4 of 5.0) in clinical practice, with an overall satisfaction score of 4.1 of 5.0 using a 5-point Likert scale questionnaire (Figure 3B; eTable 12 in Supplement 2).

## Discussion

In this RCT, our results suggest that the iNCDSS is effective and safe in hospitalized patients with T2D. The dynamic insulin titration by iNCDSS led to a progressive decrease in mean sensor glucose concentration during intervention, with the TIR (70-180 mg/dL) achieving the guideline-recommended target^23^ and being noninferior to endocrinologists. The safety of the iNCDSS was further supported by its numerically lower rates of hypoglycemia and hyperglycemia, with no reported severe glycemic events. Importantly, physicians widely accepted the iNCDSS’s advice, expressing satisfaction with its clear interface, effective and safe dosage recommendations, and the time it saved. These clinical outcomes, along with high physician adherence, demonstrated iNCDSS’s clinical utility. Subgroup analyses identified that the iNCDSS remained effective in participants across common subcutaneous insulin regimens, including once-daily basal, basal-bolus, and premixed regimens. Additionally, no significant between-group differences were observed in the insulin-experienced and insulin-naive subgroups.

Optimal insulin therapy is crucial for improving glycemic control and reducing complications for T2D. Despite the emergence of digital insulin-titration technologies, few are implemented in clinical practice. A rule-based glycemic management system for basal-bolus insulin titration (GlucoTab [decide Clinical Software GmbH]) has been used in hospitalized patients with T2D. A pilot, single-arm, intervention study^24^ showed that this system achieved a mean sensor glucose of 151.2 mg/dL during a 4- to 10-day intervention, but its generalization to other sites remains limited. The iNCDSS incorporated more patient-specific features into the advanced AI-based algorithm, generating personalized insulin recommendations. Moreover, it can be integrated into workflows of different hospitals according to standardized protocol, and our multicenter RCT demonstrated its efficacy in optimizing glycemic control with a mean TIR of 76.4% during 5-day intervention, comparable with endocrinologists. Besides basal-bolus regimens, the iNCDSS also supports premixed insulin titration, which is commonly used in outpatient settings. An AI-driven application designed for outpatient glycemic optimization (d-Nav [Hygieia LLC]) supports weekly insulin titration for T2D by assessing glucose readings and patterns. A study showed that d-Nav could assist health care professionals in insulin titration and lead to superior glycemic control compared with health care professionals alone in patients with T2D.^25^ However, its interval for insulin titration was 1 to 4 weeks, and there is no evidence of its ability to optimize blood glucose in the short term. The patients with uncontrolled T2D included in our study received real-time insulin titration and achieved the glucose target in a relatively short period, suggesting iNCDSS’s capacity for effective and timely insulin adjustments, which would greatly benefit patients needing rapid glycemic control, especially inpatients. Closed-loop insulin delivery systems, which continuously adjust insulin pumps based on CGM readings, were initially designed for type 1 diabetes.^26^ Although previous clinical trials have demonstrated its improvements in glucose control for T2D,^27,28,29^ its universal applicability is hindered by barriers such as patient acceptance, cost, and device complexity.^30,31,32^ The iNCDSS, which leverages point-of-care capillary blood glucose measurements and encompasses common subcutaneous insulin regimens, would be more suitable for patients with T2D in clinical practice.

The iNCDSS is an AI-driven tool that uses electronic health records incorporating typical characteristics of the general T2D population to output real-time and personalized insulin titration covering the most commonly used subcutaneous insulin regimens in T2D.^19^ Suboptimal glycemic control for inpatients with T2D often results in prolonged hospital stays and poor clinical outcomes,^33^ whereas the iNCDSS offers a pragmatic solution to optimal insulin titration. In our study, the iNCDSS showed noninferiority compared with endocrinologists in glycemic control optimization in hospitalized patients with uncontrolled T2D. The patients included in our study also presented with chronic comorbidities, indicating our system’s adaptability to general wards to facilitate optimized glycemic control. Moreover, physicians’ high adherence and positive feedback further underscore the system’s potential to alleviate clinical burden in general wards. Inpatient glycemic management is additionally complicated by acute illness and stress-induced hyperglycemia. Because the iNCDSS could provide dynamic and timely basal-bolus insulin adjustments, it may be applicable for managing hyperglycemia in acutely ill patients with pronounced glucose fluctuations, although further validation is needed. Furthermore, the iNCDSS’s ability to support precise and frequent insulin titration based on capillary blood glucose measurements is also critical for discharged patients, indicating its potential as a scalable solution for glucose management in outpatient settings with further algorithm update and hardware device integration.

### Strengths and Limitations

There are many strengths of our study. We conducted an RCT to assess an AI-based clinical decision-making system that leverages high-quality electronic health record datasets and machine learning algorithms for real-time insulin dosage recommendations. Additionally, the performance of the iNCDSS demonstrates noninferiority compared with senior endocrinologists who represent the highest level of expertise in clinical practice, suggesting promise for addressing the need for optimization of glycemic control in patients with T2D. Furthermore, the integration of the iNCDSS into clinical workflows facilitates its ease of use in an inpatient setting, potentially accelerating its adoption for future widespread clinical applications. Furthermore, our multicenter study design allowed us to assess the safety and efficacy of the iNCDSS tool across different health care systems and patient populations. This supports the generalizability of our findings and further establishes the system’s potential for widespread use in managing T2D.

This study also has limitations. First, although our study is a multicenter RCT, all participating sites were Chinese hospitals, potentially limiting the generalizability of our findings to patients of other ethnicities. Second, the trial was conducted during a relatively short period, which may not reflect glycemic control over longer durations. Third, our study was conducted in specialized endocrinology wards, focusing on patients with uncontrolled T2D admitted primarily for optimization of glycemic control, and did not account for patients with stress-induced hyperglycemia, medical nutrition support, or corticosteroid treatment. Additional studies assessing the iNCDSS for a more extended period and broader clinical application scenarios are necessary to validate its efficacy and safety.

## Conclusions

In this RCT of an AI-based iNCDSS, we developed a system that provided timely and personalized insulin dosage titration recommendations. The system demonstrated efficacy and safety in insulin dosage titration, showing noninferiority compared with the performance of senior physicians in treating hospitalized patients with T2D in specialized endocrinology wards. Additional studies are needed to evaluate the efficacy and safety of the iNCDSS in specific patient cohorts to facilitate its widespread adoption by health care systems.

## Notes

Supplement 1.
Trial Protocol

Supplement 2. **eTable 1.** Demographics and Baseline Characteristics of the Randomized Population, and Participants With/Without Primary Outcome **eTable 2.** Demographics and Baseline Characteristics of the Randomized Population **eTable 3.** Baseline Characteristics by Site **eTable 4.** Demographics and Baseline Characteristics of the Per-Protocol Population **eTable 5.** Glucose Control and Mean Daily Insulin Dose in the Per-protocol Population **eTable 6.** Sensitivity Analyses **eTable 7.** Insulin Regimens and Glucose Control **eTable 8.** Prior History of Insulin Use and Glucose Control **eTable 9.** Baseline HbA1_c_ and Glucose Control **eTable 10.** Main Outcomes During the Night (0000-0800 h) and Daytime (0800-0000h) **eTable 11.** Safety Findings **eTable 12.** Healthcare Professional Post-Intervention Survey Responses: Physicians Who Used the iNCDSS During the Study (n = 10)
eMethods.

Supplement 3.
Data Sharing Statement
