# Supplementary material for: Real-Time AI-Assisted Insulin Titration System for Glucose Control in Patients With Type 2 Diabetes: A Randomized Clinical Trial
Source: JAMA Netw Open. 2025 May 7;8(5):e258910. doi: 10.1001/jamanetworkopen.2025.8910 (PMC12059970; doi:10.1001/jamanetworkopen.2025.8910)
Supplement: Supplement 1. — Trial Protocol [file jamanetwopen-e258910-s001.pdf]

Study protocol

Table of Contents

1. Background 2
2. Study Objectives 2
3. Study Design 3
4. Safety and Adverse Events 6
5. Participant Termination 7
6. Statistical Analyses 7
7. Ethical Requirement 8
8. Study Confidentiality 8
9. Training of Research Staff 8
10. Reference 8

1. Background

Type 2 diabetes is prevalent and poses a great burden in the world. Data from the International Diabetes Federation (IDF) showed that the number of diabetic patients had reached 451 million in 2017, and it will rise to 693 million by 2045[1]. While management of hyperglycemia in well-targeted range can markedly improve diabetic complications and reduce mortality in diabetic patients[2, 3]. Many type 2 diabetic patients require insulin therapy for glycemic control in the course of disease progression. However, two-thirds of insulin users sustain glycated hemoglobin (HbA1c) levels above 7.0% (53 mmol/L) and more than a third sustain HbA1c levels higher than 9.0% (75 mmol/L) mainly due to inaccurate and inertia titration of insulin dosage[4].

Effective and safe insulin therapy is essential for glycemia management in type 2 diabetic patients. The glycemic targets achieved respond to acute illness, changes in the amounts and timing of dietary intake, physical exercise and anti-diabetic medication etc.[5]. Moreover, conventional insulin therapy increases the risk of hypoglycemia, which is also associated with increased morbidities and length of hospital stay[6]. Therefore, personalized and frequent dosage titrations are indispensable to overcome constant variations in insulin requirements. The glycemia management in type 2 diabetes patients has long been hindered by the need for experienced physicians to make insulin dosage titration. Consequently, glycemic control is often inadequate. To achieve this goal, an accurate and real-time insulin dosage titration system is needed at an individual level. Of note, current tools are mostly created based on general guidelines and cannot meet the need for clinicians to precisely titrate insulin dosage[7].

Artificial intelligence (AI) approaches have emerged as potentially powerful tools to mine electronic health records (EHRs) data to aid in disease diagnosis and management [8-10], mimicking and perhaps even augmenting the physicians in clinical decision-making. The application of artificial intelligence has been involved in image recognition of diabetic retinopathy and risk prediction for diabetes[11]. The recent promising work on EHRs-derived modelling[12] suggests that the incorporation of machine learning may enable clinical decisions of insulin dosage titration.

In the previous study, we have built and validated an artificial intelligence-based insulin clinical decision support system (iNCDSS) based on the data retrospectively obtained from the EHRs of hospitalized type 2 diabetic patients who received insulin therapy from January 2013 to October 2018 in the Department of Endocrinology and Metabolism, ZhongShan Hospital, Shanghai, China. After the EHR notes were annotated using a deep NLP information extraction model, we used a machine-learning method, eXtreme Gradient Boosting (XGBoost) to derive an algorithm that provides insulin dosage recommendations. The model performed well with a mean absolute relative difference (MARD)<10%. In the present study, we conduct a multicenter, randomized controlled trial to evaluate the efficacy and safety of iNCDSS system on glycemic control in type 2 diabetic patients who received insulin therapy in the clinical setting.

2. Study Objectives

The objective of the study is to evaluate the efficacy and safety of insulin dosage titration by the iNCDSS system as compared with senior physicians in glucose control in type 2 diabetic patients treated with subcutaneous insulin injection. We hypothesize that the glycemic control in type 2 diabetic patients receiving insulin dosage titrated according to the iNCDSS system recommendation is non-inferior to that in patients receiving insulin dosage titrated by senior physicians assessed by the percentage of time of sensor glucose concentration in the targeted range.

2.1 Primary Objectives

To evaluate the effect of insulin dosage titration according to iNCDSS system on glucose control assessed by percentage of time of sensor glucose measurement in target range (TIR) of 3.9-10.0 mmol/L during the 5 days trial period as compared to senior physicians in type 2 diabetic patients with insulin therapy.

2.2 Secondary Objectives

(1) To evaluate the effect of insulin dosage titration according to iNCDSS system on glucose control assessed by the percentage of time of sensor glucose concentration above range (10.1-13.9 mmol/L or >13.9 mmol/L) or below range (3.0-3.8 mmol/L or <3.0 mmol/L) during the 5 days trial period as compared to senior physicians in type 2 diabetic patients with insulin therapy.

(2) To evaluate the effect of insulin dosage titration according to iNCDSS system on mean sensor glucose concentration, glucose management indicator (GMI), and glycemic variability (%CV) during the 5 days trial period as compared to senior physicians in type 2 diabetic patients with insulin therapy.

(3) To evaluate the effect of insulin dosage titration according to iNCDSS system on mean pre-meal and pre-bed capillary glucose concentration at each defined time during the 5 days trial period as compared to senior physicians in type 2 diabetic patients with insulin therapy.

(4) To evaluate the risk of hypoglycemia of insulin dosage titration according to iNCDSS system as compared to senior physicians in type 2 diabetic patients with insulin therapy.

(5) To compare the mean total daily insulin dose in type 2 diabetic patients receiving insulin titration by iNCDSS system and by senior physicians.

3. Study Design

3.1 Design Overview

The study is a multicenter, patient-blind, and randomized controlled trial to evaluate the efficacy and safety of iNCDSS in the clinical application. Eligible patients are randomly allocated into two groups at a ratio of 1:1 after screening and run-in period. Randomization is stratified according to sites and baseline HbA1c level. Patients in the intervention group receive insulin dosage titration set by the iNCDSS assisted system and patients in the control group receive insulin dosage titration set by senior physicians. A total of 142 type 2 diabetic patients will be enrolled and this study will be conducted in the wards of Department of Endocrinology and Metabolism in Zhongshan Hospital, Xuhui Central Hospital, and Shanghai Fifth People's Hospital, Fudan University. All patients are studied for 5 consecutive days. Continuous glucose monitoring (CGM) is performed using flash glucose monitoring (Abbott Freestyle Libre, USA) to measure the primary outcome of the percentage of time of sensor glucose concentration in the target range during the 5 days trial period. The CGM data will be analyzed retrospectively, and the treatment will not be influenced by data gained by CGM.

3.2 Study Population

Eligible patients should meet the inclusion criteria and be excluded from the study if they meet any of the exclusion criteria.

3.2.1 Inclusion criteria

Patients included in this study should meet the following criteria:

1) Patients with type 2 diabetes;
2) Aged ≥18 years old;
3) Treated with diet alone, any combination of oral antidiabetic agents, and/or insulin

therapy for at least 3 months;

4) HbA1c: 7.0%-11.0%.

3.2.2 Exclusion criteria

Patients will be excluded from this study if they meet any of the following exclusion criteria:

1) Acute diabetic complications including diabetic ketoacidosis or hyperglycemic hyperosmolar state;
2) BMI≥45 kg/m^2;
3) Women who are pregnant or breast-feeding;
4) Subjects with severe cardiac, hepatic, or renal diseases;
5) Subjects with any psychiatric or psychological diseases;
6) Subjects with severe oedema, infections, or peripheral circulation disorders;
7) Patients plan to receive surgery during hospitalization;
8) Subjects who cannot comply with the protocol.

3.3 Randomization and masking

3.3.1 Randomization

Randomization will be conducted by the statistician of Department of Statistics, Public Health and Preventive Medicine, Fudan University, and generated by SAS software. The randomization scheme is generated and concealed until an eligible participant is ready to be randomized. Prior to randomization, the study coordinator should confirm that all screening procedures have been completed, the participant meets all eligibility criteria, and all required baseline data have been collected. Randomization is stratified according to sites and HbA1c levels (<8.0% or ≥8.0%) and eligible patients are randomly allocated into intervention and control groups at a ratio of 1:1.

3.3.2 Masking

Treatment allocation will be masked from participants and be concealed with sealed opaque envelopes. Although this is a single-blinded trial, the research assistants who collect study outcome data will be masked to participants' intervention assignment. In addition, the adjudicators for end-points will not be aware of study-group assignments.

3.4 Study Procedure

The study consists of the following periods: 1) a screening period before admission (<2 weeks); 2) a run-in period on the first day of hospitalization; and 3) a 5-days intervention period during hospitalization (Figure 1).

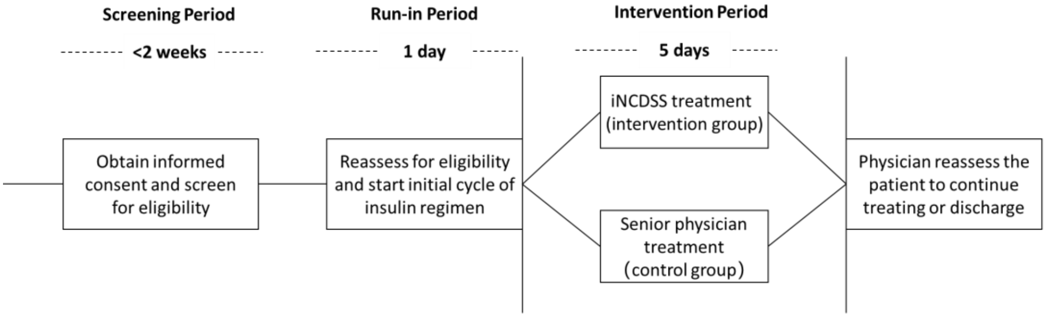

Figure 1. Flow chart

3.4.1 Screening period

T2D patients will be eligible to be screened in the study. The information about the study will be provided to patients in oral and in writing. Written informed consent for participation in the study is

obtained before performing any study-specific screening tests or evaluations. After signing the informed consent form, patients will be assigned a screening number and assessed for eligibility. Clinical characteristics and blood HbA1c level are evaluated during the screening period. The screening period will be completed until admission.

3.4.2 Run-in period

The first day of hospitalization is the run-in period. Patients are reassessed for eligibility with regard to HbA1c results. Height, body weight, HbA1c level, and previous diabetes treatments are recorded. In the run-in period, the initial insulin regimen and dose are determined by the physician based on guidelines and patient's disease status. The iNCDSS system fetches the patient’s clinical information and initial insulin regimen and dosage and then recommends the next insulin dose. All obligatory assessments should be completed in this period. The data obtained from clinical evaluation and laboratory tests for screening are used for baseline assessment as well. After the run-in period, subjects will be randomly assigned to the intervention group and the control group at 1:1 ratio.

3.4.3 Intervention period

3.4.3.1 Intervention group

The iNCDSS system is an artificial intelligence-based insulin clinical decision support system using an artificial intelligence-based system to extract clinically relevant features from EHR notes to mimic the insulin dosage titration by senior endocrinology physicians. The iNCDSS is installed in the doctor’s advice interface of the health information system (HIS) to real-time read patient information and provide insulin dosage regimens. In the intervention group, insulin dosage is modified and titrated according to the iNCDSS recommendation. The physicians can review the current recommendation and choose “accept” or “reject”. The nurse staff will conduct insulin injection according to the doctor prescribed. To assess compliance, physicians’ adherence to the recommended insulin dosage will be documented and any deviation from recommended dosage to administered insulin dosage will be recorded.

3.4.3.2 Control group

Patients in the control group receive insulin dosage titration set by senior physicians based on SMBG and clinical information.

3.5 Study Outcomes

3.5.1 Primary outcomes

The percentage of time of sensor glucose measurements in targeted range (3.9-10 mmol/L) during the 5-days trial period.

3.5.2 Secondary outcomes

1. The percentage of time of sensor glucose measurement above range (10.1-13.9 mmol/L) during the 5 days trial period;
2. The percentage of time of sensor glucose measurement above range (>13.9 mmol/L) during the 5 days trial period;
3. The percentage of time of sensor glucose measurement below range (3.0-3.8 mmol/L) during the 5 days trial period;
4. The percentage of time of sensor glucose measurement below range (<3.0 mmol/L) during the 5 days trial period;
5. The mean sensor glucose concentration;

6. The glucose management indicator (GMI);

7. The glycemic variability (%CV);

8. The mean pre-meal and pre-bed capillary glucose concentration at each defined time based on SMBG;

9. The risk of incidence of hypoglycemia events;

10. The risk of incidence of hyperglycemic events;

11. The mean total daily insulin dose.

4 Safety and Adverse Events

4.1 Definition of Adverse Events

The included hospitalized T2D patients with insulin therapy might develop hypoglycemia, hyperglycemia events with/without diabetic ketoacidosis or with/without hyperosmotic state, or other adverse events during the trial period.

4.1.1 Hypoglycemia

We determine the number of patients with hypoglycemia as defined by the Joint Position Statement of the American Diabetes Association and the European Association for the Study of Diabetes[13, 14]. Minor hypoglycemia is defined as a recorded capillary blood glucose level <3.9 mmol/L, which the patient has been able to self-treat. Clinical significant hypoglycemia is defined as a recorded capillary blood glucose level <3.0 mmol/L. Severe hypoglycemia events are defined as a capillary glucose level < 2.2 mmol/L or an episode that requires of assistance of another person due to severe impairment in consciousness or behavior.

4.1.2 Acute hyperglycemia complications of type 2 diabetes

Capillary blood glucose >20.0 mmol/L, and/or ketoacidosis, and/or hyperosmotic status.

4.1.3 Other Adverse Events

An adverse event can be any unfavorable and unintended sign (e.g. an abnormal laboratory finding), symptom (for example nausea, chest pain), or disease temporally associated with the use of a medical product, whether considered related to the medical product.

4.1.4 Severe adverse events

According to the definition by the Food and Drug Administration, USA, severe adverse events are defined as any of the following:

- Life-threatening experience
- Death
- Prolongation of existing hospitalization
- A persistent or significant disability/incapacity
- A congenital anomaly/birth defect

4.2 Recording and Reporting of Adverse Events

All non-serious and serious adverse events will be collected from the time of signature of informed consent until the end of the trial and recorded in the case report form. The occurrence time, clinical manifestation, treatment, duration, and outcome should be recorded in detail in the case report form. If an abnormal laboratory examination occurs, the patient should be followed up until the results of the examination return to normal, or to the level before treatment, or to determine if it is not related to the treatment.

If severe adverse events occur in participants, physicians should immediately provide the appropriate care to ensure their safety. Additionally, physicians will report the adverse events and

treatment to the Principal Investigator and the Committee within 24 hours and complete the report form.

4.3 Safety Monitoring

In the course of the study, an expert committee will be set up, which consists of two chief physicians who are independent of the two groups. The expert committee will review the trial program and adverse events and make a decision of continuing or suspension of the study. If the patient develops severe hyperglycemia events (capillary blood glucose >20.0mmol/L), or clinical significant and severe hypoglycemia (capillary blood glucose <3.0mmol/L), the expert committee will review the regimen provided by the iNCDSS. If the recommendation of iNCDSS system is consistent with that of the expert committee, patients continue to be treated in accordance with the insulin dosage titration recommended by the iNCDSS; else if the expert committee does not endorse the insulin dosage titration recommended by the iNCDSS, then the patient will be judged to withdraw from the study and receive treatment by physicians. If patients suffer from severe events such as ketoacidosis, and/or hypertonic coma, the patients will be judged to terminate the study and receive an intravenous infusion of insulin treatment. And if patients suffer from hypoglycemia coma, the patients will be judged to terminate the study and receive an intravenous infusion of glucose therapy.

All case report forms for each participant should be filled out by study staff in a timely manner. The case report form should be double-checked for potential errors or missing data prior to patients leaving the clinic. Original documents and case report forms will be stored in the study office. All data will be double-entered by researcher staff. Whenever inconsistencies are found, the data will be corrected by re-examination of the original case report forms or laboratory reports.

5 Participant Termination

Patients may be terminated in the following situations:

1) Patients request to withdraw from the study;

2) The expert committee suggests terminating the study for a medical perspective;

3) Adverse events or other unexpected reasons.

At any time, patients are free to discontinue or withdraw from the study, without prejudice for further treatment. A patient who decides to withdraw from the study will always be asked about the reason(s) and the presence of any adverse events. If possible, they will be seen and assessed by an investigator(s). Adverse events will be followed up.

6 Statistical Analyses

6.1 Sample Size Estimate

The design of this study is a non-inferiority randomized clinical trial to explore the efficacy and safety of the iNCDSS compared with senior physicians. The sample size calculation was based on the primary outcome. According to our previous study, the proportion of time the sensor glucose measurement in the target glucose range of 3.9–10·0 mmol/L during the intervention period was 80% with a standard deviation of 0.12. A margin of 6 percentage points was calculated to assess non-inferiority in the difference in the TIR between the groups. We calculated that 142 patients were needed (considering a dropout rate of approximately 10%) to achieve a power of 80% with an α value of 0·025 (one-sided 95% CI).

6.2 Data Analysis and Interpretation

Values are presented as mean (SD), median (IQR) or number (%), unless stated otherwise. All analyses are performed by intention-to-treat, and all patients who have available CGM data are

included in the primary analysis and all secondary analyses unless otherwise noted. All efficacy outcomes and continuous glucose monitoring based outcomes are summarized and analyzed using the intervention period and intention-to-treat analysis set, and safety analyses included all data up to the end of the study. The primary and secondary outcomes are compared between the two groups with a linear mixed-effect regression model. Missing data are not imputed. The noninferiority hypothesis is tested for the primary outcome by estimating the difference in the percentage of sensor glucose measurement in TIR between the iNCDSS group and the physician group, and the lower limit of 95% CI of -6.0% is estimated as the noninferiority threshold. The proportion of participants with events (capillary glucose concentrations <3.0 mmol/L, <2.2 mmol/L, >20.0mmol/L, and/or ketoacidosis) in each group is compared with Fisher's exact test. Compliance is calculated as the percentage of the number of insulin orders where dosages are actually ordered according to iNCDSS recommendation against the total number of insulin orders. All statistical analyses are performed with the use of SAS 9.3 software and R software.

7 Ethical Requirement

This study protocol will be reviewed by the Ethics Committee of the Zhongshan Hospital of Fudan University, Xuhui Central Hospital, and Shanghai Fifth People's Hospital, Fudan University. The study will comply with the declaration of Helsinki and Chinese laws and regulations on clinical trials. The study will respect the rights of participants, and written informed consent will be obtained. The study will be registered at www.clinicaltrials.gov.

8 Study Confidentiality

The results of the study may be published in a medical journal, and we ensure the patient's personal information will not be leaked according to the relevant legal requirements.

9 Training of Research Staff

Before the first patient is entered into the study, a principal investigator will review and discuss the requirements of the Clinical Study Protocol and related documents with the investigational staff, and train them in any study specific procedures. The principal investigator will ensure that appropriate training relevant to the study is given to all these staff, and that any new information relevant to the performance of this study is forwarded to the staff involved. The Principal Investigator will maintain a record of all individuals involved in the study (medical, nursing and other staff).

10 Reference

1. Cho NH, Shaw JE, Karuranga S, et al. IDF Diabetes Atlas: Global estimates of diabetes prevalence for 2017 and projections for 2045. Diabetes Res Clin Pract. Apr 2018;138:271-281. doi:10.1016/j.diabres.2018.02.023
2. Stratton IM, Adler AI, Neil HA, et al. Association of glycaemia with macrovascular and microvascular complications of type 2 diabetes (UKPDS 35): prospective observational study. Bmj. Aug 12 2000;321(7258):405-12. doi:10.1136/bmj.321.7258.405
3. Holman RR, Paul SK, Bethel MA, Matthews DR, Neil HA. 10-year follow-up of intensive glucose control in type 2 diabetes. N Engl J Med. Oct 9 2008;359(15):1577-89. doi:10.1056/NEJMoa0806470
4. Nawaz MS, Shah KU, Khan TM, et al. Evaluation of current trends and recent development in insulin therapy for management of diabetes mellitus. Diabetes Metab Syndr. Dec 2017;11 Suppl 2:S833-s839. doi:10.1016/j.dsx.2017.07.003

5. Bashan E, Herman WH, Hodish I. Are glucose readings sufficient to adjust insulin dosage? Diabetes Technol Ther. Jan 2011;13(1):85-92. doi:10.1089/dia.2010.0112
6. Akirov A, Grossman A, Shochat T, Shimon I. Mortality Among Hospitalized Patients With Hypoglycemia: Insulin Related and Noninsulin Related. J Clin Endocrinol Metab. Feb 1 2017;102(2):416-424. doi:10.1210/jc.2016-2653
7. Davidson MB. How our current medical care system fails people with diabetes: lack of timely, appropriate clinical decisions. Diabetes Care. Feb 2009;32(2):370-2. doi:10.2337/dc08-2046
8. Bi WL, Hosny A, Schabath MB, et al. Artificial intelligence in cancer imaging: Clinical challenges and applications. CA Cancer J Clin. Mar 2019;69(2):127-157. doi:10.3322/caac.21552
9. Esteva A, Kuprel B, Novoa RA, et al. Dermatologist-level classification of skin cancer with deep neural networks. Nature. Feb 2 2017;542(7639):115-118. doi:10.1038/nature21056
10. Kermany DS, Goldbaum M, Cai W, et al. Identifying Medical Diagnoses and Treatable Diseases by Image-Based Deep Learning. Cell. Feb 22 2018;172(5):1122-1131.e9. doi:10.1016/j.cell.2018.02.010
11. Rigla M, García-Sáez G, Pons B, Hernando ME. Artificial Intelligence Methodologies and Their Application to Diabetes. J Diabetes Sci Technol. Mar 2018;12(2):303-310. doi:10.1177/1932296817710475
12. Liang H, Tsui BY, Ni H, et al. Evaluation and accurate diagnoses of pediatric diseases using artificial intelligence. Nat Med. Mar 2019;25(3):433-438. doi:10.1038/s41591-018-0335-9
13. Umpierrez GE, Hellman R, Korytkowski MT, et al. Management of hyperglycemia in hospitalized patients in non-critical care setting: an endocrine society clinical practice guideline. J Clin Endocrinol Metab. Jan 2012;97(1):16-38. doi:10.1210/jc.2011-2098
14. Glucose Concentrations of Less Than 3.0 mmol/L (54 mg/dL) Should Be Reported in Clinical Trials: A Joint Position Statement of the American Diabetes Association and the European Association for the Study of Diabetes. Diabetes Care. Jan 2017;40(1):155-157. doi:10.2337/dc16-2215
