# Supplementary material for: Real-Time AI-Assisted Insulin Titration System for Glucose Control in Patients With Type 2 Diabetes: A Randomized Clinical Trial
Source: JAMA Netw Open. 2025 May 7;8(5):e258910. doi: 10.1001/jamanetworkopen.2025.8910 (PMC12059970; doi:10.1001/jamanetworkopen.2025.8910)
Supplement: Supplement 2. — eTable 1. Demographics and Baseline Characteristics of the Randomized Population, and Participants With/Without Primary Outcome eTable 2. Demographics and Baseline Characteristics of the Randomized Population eTable 3. Baseline Characteristics by Site eTable 4. Demographics and Baseline Characteristics of the Per-Protocol Population eTable 5. Glucose Control and Mean Daily Insulin Dose in the Per-protocol Population eTable 6. Sensitivity Analyses eTable 7. Insulin Regimens and Glucose Control eTable 8. Prior History of Insulin Use and Glucose Control eTable 9. Baseline HbA1c and Glucose Control eTable 10. Main Outcomes During the Night (0000-0800 h) and Daytime (0800-0000h) eTable 11. Safety Findings eTable 12. Healthcare Professional Post-Intervention Survey Responses: Physicians Who Used the iNCDSS During the Study (n = 10) eMethods. [file jamanetwopen-e258910-s002.pdf]

Supplemental Online Content

Ying Z, Fan Y, Chen C, et al. Real-time AI-assisted insulin titration system for glucose control in patients with type 2 diabetes: a randomized clinical trial. *JAMA Netw Open*. 2025;8(5):e258910. doi:10.1001/jamanetworkopen.2025.8910

eTable 1. Demographics and baseline characteristics of the randomized population, and participants with/without primary outcome
eTable 2. Demographics and baseline characteristics of the randomized population
eTable 3. Baseline characteristics by site
eTable 4. Demographics and baseline characteristics of the per-protocol population
eTable 5. Glucose control and mean daily insulin dose in the per-protocol population
eTable 6. Sensitivity analyses
eTable 7. Insulin regimens and glucose control
eTable 8. Prior history of insulin use and glucose control
eTable 9. Baseline HbA1c and glucose control
eTable 10. Main outcomes during the night (0000-0800 h) and daytime (0800-0000h)
eTable 11. Safety findings
eTable 12. Healthcare professional post-intervention survey responses: physicians who used the iNCDSS during the study (n=10)
eMethods

This supplemental material has been provided by the authors to give readers additional information about their work.

© 2025 Ying Z et al. *JAMA Network Open*.

eTable1. Demographics and baseline characteristics of the randomized population, and participants with/without primary outcome.

Characteristic	Randomized population (n=149)	Participants with primary outcome (n=144)	Participants without primary outcome (n=5)
Sex, No. (%)			
Male	84 (56.4)	81 (56.2)	3 (60.0)
Female	65 (43.6)	63 (43.8)	2 (40.0)
Age, mean (SD), years	64.2 (12.0)	64.2 (12.0)	64.0 (12.6)
BMI, mean (SD), kg/m^2	25.1 (3.9)	25.2 (3.8)	21.9 (3.2)
HbA1c, mean (SD), %	9.0 (1.1)	9.0 (1.1)	9.7 (0.9)
Duration of diabetes, mean (SD), years	14.5 (9.1)	14.5 (9.2)	15.4 (4.6)
Previous diabetes treatments, No. (%)			
Diet alone	1 (0.7)	1 (0.7)	0 (0.0)
Oral agents	50 (33.6)	48 (33.3)	2 (40.0)
Insulin alone	12 (8.1)	12 (8.3)	0 (0.0)
Insulin plus oral agents	86 (57.7)	83 (57.6)	3 (60.0)

The primary outcome data in population with CGM error or loss were missing. ALT, alanine aminotransferase; AST, aspartate aminotransferase; eGFR, estimated glomerular filtration rate.

© 2025 Ying Z et al. *JAMA Network Open*.

eTable2. Demographics and baseline characteristics of the randomized population

Characteristic	iNCDSS Group (n=75)	Physician Group (n=74)
Insulin, No. (%)		
Insulin-naive	30 (40.0)	21 (28.4)
Basal insulin therapy	4 (5.3)	3 (4.1)
Premixed/biphasic insulin therapy	35 (46.7)	32 (43.2)
Basal-bolus insulin	6 (8.0)	18 (24.3)
Other glucose-lowering medications, No. (%)		
Biguanides	36 (48.0)	32 (43.2)
Meglitinides	2 (2.7)	2 (2.7)
Thiazolidinediones	6 (8.0)	6 (8.1)
Alpha-Glucosidase Inhibitors	28 (37.3)	33 (44.6)
DPP-4 inhibitors	26 (34.7)	21 (28.4)
SGLT2 inhibitors	12 (16.0)	13 (17.6)
GLP-1 agonists	3 (4.0)	3 (4.1)
Fasting BG, mean (SD), mg/dL	149.7 (59.2)	155.0 (62.1)
Fasting C-peptide, mean (SD), ng/mL	1.9 (1.4)	1.7 (1.1)
ALT, mean (SD), U/L	24.5 (26.2)	22.4 (16.0)
AST, mean (SD), U/L	22.1 (21.6)	20.3 (11.6)
Creatinine, mean (SD), μmol/L	80.0 (31.0)	77.8 (36.0)
eGFR, mean (SD), ml/min/1.73m^{-2}	82.2 (23.2)	84.3 (23.6)
Hypertension, No. (%)	40 (53.3)	41 (55.4)
Coronary artery disease or congestive heart failure, No. (%)	14 (18.7)	11 (14.9)
Charlson Comorbidity Index score, median (IQR)	4 (2-5)	4 (2-5)
Baseline insulin dosage, median (IQR), units	27.0 (22.0-36.0)	30.5 (22.0-43.5)

ALT, alanine aminotransferase; AST, aspartate aminotransferase; eGFR, estimated glomerular filtration rate.

© 2025 Ying Z et al. *JAMA Network Open*.

eTable3. Baseline characteristics by site.

Characteristic	Zhongshan Hospital (n=74)	Shanghai Fifth People's Hospital (n=52)	Xuhui Central Hospital (n=23)
Sex, No. (%)			
Male	49 (66.2)	26 (50.0)	9 (39.1)
Female	25 (33.8)	26 (50.0)	14 (60.9)
Age, mean (SD), years	61.2 (11.0)	63.9 (10.5)	74.6 (12.8)
BMI, mean (SD), kg/m^2	25.3 (3.3)	25.7 (4.4)	23.1 (3.7)
HbA_{1c}, mean (SD), %	9.0 (1.1)	9.0 (1.00)	8.9 (1.2)
Duration of diabetes, mean (SD), years	13.2 (8.00)	15.4 (10.2)	16.9 (9.3)
Previous diabetes treatments, No. (%)			
Diet alone	0 (0.0)	1 (1.9)	0 (0.0)
Oral agents	16 (21.6)	23 (44.2)	11 (47.8)
Insulin alone	7 (9.5)	2 (3.8)	3 (13.0)
Insulin plus oral agents	51 (68.9)	26 (50.0)	9 (39.1)
Charlson Comorbidity Index score, median (IQR)	4 (2-5)	3 (2-4)	5 (4-6)

Age, sex, baseline BMI, previous diabetes treatments, and Charlson Comorbidity Index score were significantly different between centers ($p<0.05$). BMI, body mass index; HbA1c, glycated hemoglobin.

© 2025 Ying Z et al. *JAMA Network Open*.

eTable4. Demographics and baseline characteristics of the per-protocol population.

Characteristic	iNCDSS Group (n=68)	Physician Group (n=68)
Sex, No. (%)		
Male	39 (57.4)	38 (54.4)
Female	29 (42.6)	31 (45.6)
Age, mean (SD), years	63.5 (12.6)	65.2 (11.8)
BMI, mean (SD), kg/m^2	25.3 (4.1)	25.2 (3.6)
HbA_{1c}, mean (SD), %	9.0 (1.2)	9.0 (1.0)
Duration of diabetes, mean (SD), years	12.9 (9.1)	15.8 (8.9)
Previous diabetes treatments, No. (%)		
Diet alone	0 (0.0)	1 (1.5)
Oral agents	26 (38.2)	17 (25.0)
Insulin alone	5 (7.4)	7 (10.3)
Insulin plus oral agents	37 (54.4)	43 (63.2)

The analysis included participants who completed the trial according to the protocol. ALT, alanine aminotransferase; AST, aspartate aminotransferase; eGFR, estimated glomerular filtration rate.

© 2025 Ying Z et al. *JAMA Network Open*.

eTable5. Glucose control and mean daily insulin dose in the per-protocol population.

	iNCDSS Group (n=68)	Physician Group (n=68)	Estimated treatment difference (95%CI)	P value
Proportion of time in which the glucose level is within range, %				
70-180 mg/dL, mean (SD), %[a]	76.1 (16.5)	73.9 (16.9)	2.2 (-3.4, 7.8)	0.44
181-250 mg/dL, mean (SD), %	15.9 (11.4)	18.1(12.1)	-2.2 (-6.2, 1.8)	0.28
>250 mg/dL, mean (SD), %	4.0 (7.7)	4.1 (8.3)	-0.2 (-2.9, 2.5)	0.89
54-70 mg/dL, mean (SD), %	3.4 (4.4)	3.1(5.0)	0.3 (-1.3, 1.9)	0.73
<54 mg/dL, median (IQR), %	0.0 (0.0-0.5)	0.0 (0.0-0.6)	-0.1 (-0.8, 0.6)	0.75
Mean glucose, mean (SD), mg/dL	139.9 (27.7)	144.2 (27.7)	-4.3 (-13.7,5.0)	0.36
CV of glucose, mean (SD), %	31.5 (6.9)	30.3(6.6)	1.2 (-1.0, 3.5)	0.29
GMI, mean (SD), %	6.7 (0.7)	6.8 (0.7)	-0.1 (-0.3, 0.1)	0.36
Capillary glucose concentrations, mean (SD)				
Pre-breakfast (0500-0800h), mg/dL	131.6 (28.3)	140.0 (32.0)	-8.9 (-18.9,1.2)	0.09
Pre-lunch (1100-1300h), mg/dL	152.1 (48.9)	155.7 (46.7)	-2.0 (-16.0,12.0)	0.78
Pre-dinner (1600-1800h), mg/dL	168.6 (44.3)	179.3 (47.1)	-9.9 (-24.9,5.1)	0.20
Pre-bed (2000-2200h), mg/dL	167.6 (48.4)	173.8 (45.2)	-4.4 (-19.3, 10.5)	0.56
Mean daily insulin dose, median (IQR), units	27.4 (20.6-35.1)	30.2 (23.1-39.3)	-4.8 (-8.7, -0.9)	0.02

The analysis included participants who completed the trial according to the protocol. The primary and secondary outcomes were compared between the two groups with a linear mixed-effect regression model, which included treatment (iNCDSS and senior physician group) as a fixed effect, site as a random effect, and baseline HbA1c level as a covariate. Abbreviations: CV, coefficient of variation; GMI, glucose management indicator. [a] Primary outcome.

© 2025 Ying Z et al. *JAMA Network Open*.

eTable6. Sensitivity analyses

A. Included baseline dosage in model

Outcome	**Estimated treatment difference (95%CI) [P value]**
Proportion of time the glucose level in 70-180 mg/dL[a]	2.3 (-3.2, 7.8) [0.41]
Mean daily insulin dose	-1.2 (-2.9, 0.6) [0.19]

All randomized population (the intention to treat population) was used for analysis. The primary and secondary outcomes were compared between the two groups with a linear mixed-effect regression model, which included treatment (iNCDSS and senior physician group) as a fixed effect, site as a random effect, and baseline HbA1c level and baseline insulin dosage as covariates. Abbreviations: [a] Primary outcome.

B. Multiple Imputation

Outcome	**Estimated treatment difference (95%CI) [P value]**
Proportion of time the glucose level in 70-180 mg/dL	2.6 (-2.9, 8.1) [0.39]

We used multiple imputation for missing primary outcome data, using baseline characteristics (age, sex, body mass index, diabetes duration, HbA1c, fasting BG, fasting C-peptide, ALT, AST, Creatinine, eGFR), the treatment indicator, and sites to generate 5 imputed data sets. Each of the 5 datasets were used to analyze the primary outcome with the linear mixed-effect regression model, which included treatment (iNCDSS and senior physician group) as a fixed effect, site as a random effect, and baseline HbA1c level as a covariate. The estimated coefficients and standard errors from the 5 models were combined into a final estimated coefficient and standard error.

© 2025 Ying Z et al. *JAMA Network Open*.

eTable7. Insulin regimens and glucose control.

	Basal insulin regimen (N=19)		Premixed/ biphasic insulin regimen (N=100)		Basal bolus insulin regimen (N=30)	
	iNCDSS (n=15)	Physician (n=4)	iNCDSS (n=50)	Physician (n=50)	iNCDSS (n=10)	Physician (n=20)
Proportion of time in which the glucose level is within range, %						
70-180 mg/dL, mean (SD), %[a]	78.6 (17.8)	65.0 (40.2)	74.7 (17.0)	74.5 (14.9)	81.1 (9.7)	73.3 (15.0)
181-250 mg/dL, mean (SD), %	13.2(12.3)	16.6 (15.4)	17.5 (11.4)	18.3 (11.7)	10.6 (7.2)	18.5 (12.9)
>250 mg/dL, mean (SD), %	2.6 (5.4)	15.4 (28.8)	4.5 (8.8)	3.6 (5.4)	3.4 (3.6)	3.3 (4.0)
54-70 mg/dL, mean (SD), %	4.4 (6.1)	0.8 (1.6)	2.8 (3.7)	3.1 (5.0)	4.0 (4.6)	3.5 (5.4)
<54 mg/dL, median (IQR), %	0 (0-0.4)	0 (0-2.2)	0 (0-0.2)	0 (0-0.6)	0 (0-0.8)	0 (0-0.5)
Mean glucose, mean (SD), mg/dL	131.9 (24.6)	164.9 (60.8)	143.5 (29.6)	143.6 (24.6)	133.9 (18.2)	142.1 (24.6)
CV of glucose, mean (SD), %	30.8 (8.9)	22.8 (9.4)	30.8 (6.1)	30.8 (5.7)	34.1 (8.3)	31.3 (8.0)
GMI, mean (SD), %	6.5 (0.6)	7.3 (1.5)	6.7 (0.7)	6.8 (0.6)	6.5 (0.4)	6.7 (0.6)
Mean daily insulin dose, median (IQR), units	11.2 (9.1- 22.5)	20.0 (14.0- 26.1)	27.2 (21.2- 33.9)	28.0 (22.0- 35.5)	37.1 (31.4, 48.8)	45.8 (34.8, 53.1)
Capillary glucose concentrations, mean (SD)						
Pre-breakfast (0500-0800h), mg/dL	110.4(17.4)	166.4 (41.3)	136.9 (27.5)	137.9 (27.2)	132.8 (30.7)	148.2 (51.1)
Pre-lunch (1100-1300h), mg/dL	172.1(56.0)	210.1 (43.2)	147.9 (45.6)	153.9 (48.6)	134.3 (37.6)	158.4 (37.9)
Pre-dinner (1600-1800h), mg/dL	153.4(34.1)	210.0 (73.2)	175.5 (48.1)	180.3 (47.2)	150.0 (38.3)	180.2 (42.2)
Pre-bed (2000-2200h), mg/dL	189.3(47.5)	208.4 (47.4)	159.7 (50.2)	165.6 (43.0)	182.1 (35.2)	200.9 (52.9)

Abbreviations: CV, coefficient of variation; GMI, glucose management indicator. [a] Primary outcome.

© 2025 Ying Z et al. *JAMA Network Open*.

eTable8. Prior history of insulin use and glucose control.

	**Previous insulin-naïve patients (N=51)**		**Previous insulin-treated patients (N=98)**	
	iNCDSS (n=30)	Physician (n=21)	iNCDSS (n=45)	Physician (n=53)
Proportion of time in which the glucose level is within range, %				
70-180 mg/dL, mean (SD), %[a]	73.6 (16.6)	73.7 (17.4)	78.2 (16.1)	73.6 (16.7)
181-250 mg/dL, mean (SD), %	16.8 (11.1)	18.8 (14.2)	14.9 (11.6)	18.0 (11.3)
>250 mg/dL, mean (SD), %	4.4 (7.4)	4.0 (7.0)	3.7 (7.9)	4.2 (8.7)
54-70 mg/dL, mean (SD), %	4.3 (5.3)	2.6 (4.5)	2.6 (3.5)	3.3 (5.2)
<54 mg/dL, median (IQR), %	0.0(0.0-0.4)	0.0(0.0-0.0)	0.0(0.0-0.4)	0.0(0.0-0.7)
Mean glucose, mean (SD), mg/dL	141.8 (28.1)	143.9 (31.1)	138.6 (27.4)	144.6 (26.2)
CV of glucose, mean (SD), %	32.1 (7.7)	29.7 (5.4)	30.6 (6.5)	30.7 (7.3)
GMI, mean (SD), %	6.7 (0.7)	6.8 (0.7)	6.6 (0.7)	6.8 (0.6)
Mean daily insulin dose, median (IQR), units	22.8(15.5-32.5)	23.6(18.4-34.0)	30.0 (22.6-35.8)	33.0 (24.8-46.0)
Capillary glucose concentrations, mean (SD)				
Pre-breakfast (0500-0800h), mg/dL	129.4 (30.1)	136.7 (27.1)	132.2 (26.8)	144.3 (38.8)
Pre-lunch (1100-1300h), mg/dL	164.1 (42.9)	158.8 (47.1)	142.0 (49.3)	157.9 (47.3)
Pre-dinner (1600-1800h), mg/dL	170.1 (32.8)	185.6 (43.3)	166.0 (52.3)	180.4 (49.1)
Pre-bed (2000-2200h), mg/dL	188.4 (37.7)	176.4 (46.7)	155.4 (51.7)	177.2 (49.5)

Abbreviations: CV, coefficient of variation; GMI, glucose management indicator. [a] Primary outcome.

© 2025 Ying Z et al. *JAMA Network Open*.

eTable9. Baseline HbA1c and glucose control.

	**HbA1c <8% (N=37)**		**HbA1c ≥8% (N=112)**	
	iNCDSS (n=19)	Physician (n=18)	iNCDSS (n=56)	Physician (n=56)
Proportion of time in which the glucose level is within range, %				
70-180 mg/dL, mean (SD), %[a]	79.6 (13.4)	75.7 (16.7)	75.3 (17.2)	72.9 (16.9)
181-250 mg/dL, mean (SD), %	13.5 (9.7)	17.5 (12.8)	16.5 (11.8)	18.5 (11.9)
>250 mg/dL, mean (SD), %	1.5 (1.9)	3.4 (4.2)	4.8 (8.7)	4.4 (9.2)
54-70 mg/dL, mean (SD), %	4.2 (5.0)	2.5 (2.5)	3.0 (4.2)	3.3 (5.6)
<54 mg/dL, median (IQR), %	0 (0-0.9)	0 (0-1.1)	0 (0-0.3)	0 (0-0.3)
Mean glucose, mean (SD), mg/dL	132.4 (17.1)	141.8 (22.6)	142.5 (30.1)	145.3 (29.0)
CV of glucose, mean (SD), %	31.1 (7.3)	31.7 (5.6)	31.3 (7.0)	30.0 (7.2)
GMI, mean (SD), %	6.5 (0.4)	6.7 (0.5)	6.7 (0.7)	6.8 (0.7)
Mean daily insulin dose, median (IQR), units	20.2 (14.4-32.7)	26.8 (20.3-34.0)	28.2 (22.6-34.5)	31.40 (23.6-44.7)
Capillary glucose concentrations, mean (SD)				
Pre-breakfast (0500-0800h), mg/dL	122.5 (22.4)	132.6 (38.8)	134.0 (29.3)	145.3 (34.5)
Pre-lunch (1100-1300h), mg/dL	145.8 (36.6)	147.1 (49.7)	152.6 (51.0)	161.9 (45.8)
Pre-dinner (1600-1800h), mg/dL	160.3 (37.0)	175.8 (53.4)	170.2 (47.9)	183.9 (45.4)
Pre-bed (2000-2200h), mg/dL	173.3 (49.8)	165.6 (34.4)	167.0 (49.2)	180.5 (51.7)

Abbreviations: CV, coefficient of variation; GMI, glucose management indicator. a Primary outcome.

© 2025 Ying Z et al. *JAMA Network Open*.

eTable10. Main outcomes during the night (0000-0800 h) and daytime (0800-0000h).

	iNCDSS Group (n=74)	Physician Group (n=70)	Estimated treatment difference (95%CI)	P value
Overnight period (0000-0800h)				
Proportion of time in the target glucose range 70-180 mg/dL, mean (SD), %[a]	84.9 (15.1)	81.6 (17.9)	3.3 (-2.0, 8.6)	0.23
Mean glucose, mean (SD), mg/dL	119.1 (23.4)	125.5 (27.8)	-6.4 (-14.8, 1.9)	0.13
GMI, mean (SD), %	6.2 (0.6)	6.3 (0.7)	-0.2 (-0.4, 0.04)	0.13
Glucose variability, mean (SD), CV %	28.3 (9.6)	27.1 (8.6)	1.3 (-1.6, 4.1)	0.39
Daytime period (0800-000h)				
Proportion of time in the target glucose range 70-180 mg/dL, mean (SD), %[a]	72.0 (20.6)	69.6 (20.1)	2.4 (-4.2, 8.9)	0.48
Mean glucose, mean (SD), mg/dL	150.5 (32.7)	154.0 (30.6)	-3.4 (-13.7, 6.8)	0.51
GMI, mean (SD), %	6.9 (0.8)	7.0 (0.7)	-0.1 (-0.3, 0.2)	0.51
Glucose variability, mean (SD), CV %	28.6 (6.4)	28.2 (6.9)	0.4 (-1.8, 2.6)	0.73

The data of the population with available CGM data were used for analysis. Abbreviations: CV, coefficient of variation; GMI, glucose management indicator.

[a] Primary outcome.

© 2025 Ying Z et al. *JAMA Network Open*.

eTable11. Safety findings.

	iNCDSS Group (N=75)	Physician group (N=74)
Number of events with capillary glucose <54mg/dL, No.	2	5
Number of participants with capillary glucose <54mg/dL, No. (%) [a]	2(3%)	5(7%)
Number of severe hypoglycemic events, No.[b]	0	0
Number of participants with severe hypoglycemic events, No. (%) [b]	0 (0%)	0 (0%)
Number of events with capillary glucose >360mg/dL, No.	22	24
Number of participants with capillary glucose >360mg/dL, No. (%) [a]	9 (12%)	14 (19%)
Number of ketoacidosis, No.	0	0
Number of participants with ketoacidosis, No. (%)	0 (0%)	0 (0%)
Number of other adverse events, No.	0	0
Serious adverse events (not study related), No.	0	0
Non-serious adverse events (not study related), No.	1	0
Number of participants with other adverse events, No.	1 (1%)	0 (0%)

[a] There was no statistical difference between the two iNCDSS group and senior physician group (Fisher's exact test, p>0.05). [b] Severe hypoglycemia is defined as capillary glucose concentration of less than 40 mg/dL or the patient requiring assistance from another member of healthcare staff.

© 2025 Ying Z et al. *JAMA Network Open*.

eTable12. Healthcare professional post-intervention survey responses: physicians who used the iNCDSS during the study (n=10).

Item	Score (1-5)
The iNCDSS saved me time.	4.2 (0.6)
Using the iNCDSS was intuitive and simple.	4.5 (0.5)
I often encountered problems when using the AI system.	1.9 (0.6)
The iNCDSS interface was clear.	4.6 (0.5)
The suggested iNCDSS insulin adjustments were clear.	4.4 (0.5)
I found the iNCDSS to be effective.	4.0 (0.5)
I believe the AI system was safe.	4.4 (0.5)
The iNCDSS allowed for more frequent changes getting to optimal dosing more quickly.	4.2 (0.6)
The iNCDSS reinforced my confidence.	3.9 (0.6)
I believe most users can learn to use iNCDSS quickly.	4.1 (0.6)
I would like to use iNCDSS in my future practice.	4.1 (0.6)
I would recommend the AI system to other doctors.	4.2 (0.6)
The overall satisfaction with iNCDSS.	4.1 (0.6)

Data presented as mean (SD).

© 2025 Ying Z et al. *JAMA Network Open*.

eMethods

eMethod 1. AI system report. 15
eMethod 2. Insulin regimen protocol. 17
eMethod 3. Satisfaction questionnaire. 18

© 2025 Ying Z et al. *JAMA Network Open*.

eMethod 1. AI system report.

1. Overview of the iNCDSS system

Our proposed iNCDSS is an artificial intelligence-based insulin clinical decision support workflow to provide a real-time insulin dosage titration based on the personalized algorithm and guideline recommendations. Our system now supports the following three common insulin regimens, and can dynamically adjust insulin dosage:

• Regimen 1 – participants treated with a single injection of the long-acting insulin or insulin analog (i.e.,Glargine) per day;

• Regimen 2 – participants treated with twice daily biphasic insulin (i.e., Humalog® Mix 75/25, NovoLog® Mix 70/30) or premixed insulin (i.e., Humulin® 70/30, Novolin® 70/30);

• Regimen 3 – participants treated with a short- (and rapid-) acting insulin analog (i.e., Humalog®-Lispro, NovoLog®-Aspart, Apidra®-Glulisine) before each meal and are treated with a single injection of the long-acting insulin or insulin analog (i.e.,Glargine) per day.

2. Development of the AI system

To develop and validate our AI model, we utilized a large dataset of high-quality electronic health records (EHRs) of T2D patients. Patient-specific and time-varying features were extracted from the EHRs data and were used to train the personalized insulin titration model. Preclinical studies have shown the better performance of our model compared with other algorithms and junior physicians. In addition, a single-arm intervention study has demonstrated the feasibility of our iNCDSS system in optimizing personalized insulin recommendations in real-world clinical practice [1]. As the technology iterates, we have also updated the first-generation algorithm by several methods, including adding advanced insulin and glucose-lowering drugs, simplification of the input features to make the algorithm more adaptable to multi-center deployments, using supervised learning algorithms to learn from experts' experience to ensure the safety of the dose recommendations and so on. For a detailed input feature list, please refer to **Appendix Table 1**. Notably, missing some input features is acceptable.

Appendix Table 1. List of input features

**Feature types**	**Feature names**
**Capillary glucose measurements**	Capillary blood glucose
**Prescriptions**	Insulin categories
	Insulin dose
	Anti-diabetic medications categories
	Anti-diabetic medications daily dosage
**Demographic information and physical examination**	Age
	Gender
	Height
	Weight
	Body mass index
**Medical history**	Disease diagnosis
**Laboratory testing**	Alanine aminotransferase
	Aspartate aminotransferase
	Creatinine
	Fasting C-peptide
	Fasting insulin
	Glycated hemoglobin
	Ketone bodies
	LDL cholesterol
	Triglycerides
	HDL cholesterol
	Total cholesterol

4. Deployment of the AI system

In Zhongshan Hospital, the iNCDSS model was implemented in the doctor’s order interface of the HIS to build a workflow for a clinical decision support system of insulin titration in the hospital setting, which could real-time read the updated patient information and provide insulin dosage recommendation.

© 2025 Ying Z et al. *JAMA Network Open*.

Capillary glucose measurements were conducted by the nurse staff in charge and automatedly uploaded into the HIS system. The demographic information, physical examination, and disease diagnosis were extracted from the electronic medical record systems (EMR). The laboratory testing and capillary glucose measurements were obtained from the laboratory information system (LIS), while insulin and other medications were from the computerized physician order entry (CPOE) system. All data were extracted from the databases based on patients' ID and were then assembled into the data structure required by the model. The recommendations for insulin dosage were created once the features transmitted to the model and were sent to the healthcare provider for review in the Doctor's order interface. In the Xuhui Central Hospital, and Shanghai Fifth People's Hospital, the iNCDSS model was plugged into the blood glucose management software. The glucose management software itself has captured patient characteristic fields from the EMR, LIS, CPOE. Capillary glucose measurements were also automatedly uploaded into the glucose management software. All features in software are passed into the model to output insulin dosage recommendation. The deployment interface of iNCDSS is shown in **Appendix Figure 1**.

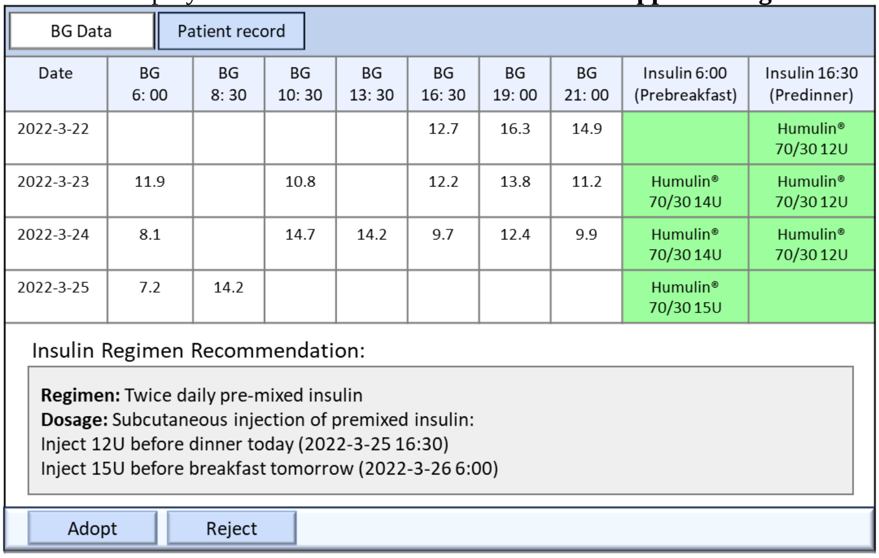

Date	BG 6: 00	BG 8: 30	BG 10: 30	BG 13: 30	BG 16: 30	BG 19: 00	BG 21: 00	Insulin 6:00 (Prebreakfast)	Insulin 16:30 (Predinner)
2022-3-22					12.7	16.3	14.9		Humulin® 70/30 12U
2022-3-23	11.9		10.8		12.2	13.8	11.2	Humulin® 70/30 14U	Humulin® 70/30 12U
2022-3-24	8.1		14.7	14.2	9.7	12.4	9.9	Humulin® 70/30 14U	Humulin® 70/30 12U
2022-3-25	7.2	14.2						Humulin® 70/30 15U	

Appendix Figure 1. Deployment interface of iNCDSS

Reference

[1] Chen, Y., et al., Real-time artificial intelligence assisted insulin dosage titration system for glucose control in type 2 diabetic patients: a proof of concept study. Current Medicine, 2023. 2(1).

© 2025 Ying Z et al. *JAMA Network Open*.

eMethod 2. Insulin regimen protocol.

1. Insulin naïve patients treated with diet, oral agents or GLP1-RAs prior to admission

- The endocrinologist decides on the initiation insulin regimen of the patient after assessing the glycemic control and the patient's wishes.
- Hold antidiabetic drugs on admission, except sulfonylureas.

1.1 Basal-bolus insulin regimen

Starting total daily insulin dose: 0.4-0.5 U/Kg/day, adjust according to insulin category and patient characteristics (including patients' BG, age and eGFR).

- Half of TDD given as long-acting(basal) insulin and half as short-acting (pre-prandial) insulin.
- Long-acting(basal) insulin given once daily, at the same time of the day.
- Short-acting(pre-prandial) insulin given in three equally divided doses before meals.

1.2 Premixed/ biphasic insulin regimen

Starting total daily insulin dose: 0.3-0.4 U/Kg/day, adjust according to insulin category and patient characteristics (including patients' BG, age and eGFR).

- Give 2 doses of premixed/biphasic insulin daily: 50%-60% of TDD before breakfast and 40%-50% before dinner.

1.3 Basal insulin regimen

Starting total daily insulin dose: 0.2-0.3 U/Kg/day, adjust according to insulin category and patient characteristics (including patients' BG, age and eGFR).

2. Patients treated with insulin prior to admission

- The endocrinologist decides on whether the patient should change the regimen after assessing glycemic control and the patient's wishes.
- Hold antidiabetic drugs on admission, except sulfonylureas.

2.1 Basal-bolus insulin regimen

- Give outpatient total daily dose (TDD) - half as long-acting(basal) insulin and half as short-acting (pre-prandial) insulin.
- Give long-acting(basal) insulin once daily, at the same time of the day
- Give short-acting (pre-prandial) insulin in three equally divided doses before meals

* If patient was on basal only therapy consider adding prandial dose (4 U, or 0.1 U/kg, or 10%, given before the main meal), according to patients' BG and meal.

2.2 Premixed/ biphasic insulin regimen

- Give outpatient TDD divided in 2 doses: 50%-60% before breakfast and 40%-50% before dinner.

2.3 Basal insulin regimen

- If patient was on basal bolus regimen, hold the original basal insulin dosage.
- If patient was on premixed/biphasic regimen, give 60%-80% of the total daily dose (TDD), and devide 50%-60% of TDD before breakfast and 40%-50% before dinner.

© 2025 Ying Z et al. *JAMA Network Open*.

eMethod 3. Satisfaction questionnaire.

If you have used this AI system in a clinical scenario, please make your selection based on the questions and options below.

Your ID Number*

____

Please select the item that best fits your situation:

1-->5 for very dissatisfied/disagree --> very satisfied/agree *

	1	2	3	4	5
The iNCDSS saved me time.	○	○	○	○	○
Using the iNCDSS was intuitive and simple.	○	○	○	○	○
I often encountered problems when using the AI system.	○	○	○	○	○
The iNCDSS interface was clear.	○	○	○	○	○
The suggested iNCDSS insulin adjustments were clear.	○	○	○	○	○
I found the iNCDSS to be effective.	○	○	○	○	○
I believe the AI system was safe.	○	○	○	○	○
The iNCDSS allowed for more frequent changes getting to optimal dosing more quickly.	○	○	○	○	○
The iNCDSS reinforced my confidence.	○	○	○	○	○
I believe most users can learn to use iNCDSS quickly.	○	○	○	○	○
I would like to use iNCDSS in my future practice.	○	○	○	○	○
I would recommend the AI system to other doctors.	○	○	○	○	○
The overall satisfaction with iNCDSS.	○	○	○	○	○

What do you think are the problems that still exist with insulin-assisted decision-making systems?

© 2025 Ying Z et al. *JAMA Network Open*.
