# Supplementary material for: Real-Time AI-Assisted Insulin Titration System for Glucose Control in Patients With Type 2 Diabetes: A Randomized Clinical Trial
Source: JAMA Netw Open. 2025 May 7;8(5):e258910. doi: 10.1001/jamanetworkopen.2025.8910 (PMC12059970; doi:10.1001/jamanetworkopen.2025.8910)
Supplement: Supplement 3. — Data Sharing Statement [file jamanetwopen-e258910-s003.pdf]

Data Sharing Statement

Ying. Real-Time AI-Assisted Insulin Titration System for Glucose Control in Patients With Type 2 Diabetes. *JAMA Netw Open*. Published May 07, 2025.
doi:10.1001/jamanetworkopen.2025.8910

Data
Additional Information: ClinicalTrials.gov Identifier: NCT04642378.
Data available: Yes
Data types: Deidentified participant data, Data dictionary
How to access data: Data Data available: Yes Data types: Deidentified participant data How to access data: The datasets generated during the study will be available from the corresponding author upon reasonable request. chen.ying4@zs-hospital.sh.cn When available: With publication Additional Information Who can access the data: Researchers whose proposed use of the data has been approved. Types of analyses: For any purpose if the proposed use of the data has been approved. Mechanisms of data availability: The datasets generated during the study will be available from the corresponding author upon reasonable request, after approval of a proposal.
When available: With publication

Supporting Documents
Document types: None

Additional Information
Who can access the data: Researchers whose proposed use of the data has been approved.
Types of analyses: For any purpose if the proposed use of the data has been approved.
Mechanisms of data availability: The datasets generated during the study will be available from the corresponding author upon reasonable request, after approval of a proposal.
